# Identification of influential spreaders in complex networks using HybridRank algorithm

**DOI:** 10.1038/s41598-018-30310-2

**Published:** 2018-08-09

**Authors:** Sara Ahajjam, Hassan Badir

**Affiliations:** Laboratory of Information and communication technologies, National School of Applied Sciences, ENSAT, Tangier, Morocco

## Abstract

Identifying the influential spreaders in complex networks is crucial to understand who is responsible for the spreading processes and the influence maximization through networks. Targeting these influential spreaders is significant for designing strategies for accelerating the propagation of information that is useful for various applications, such as viral marketing applications or blocking the diffusion of annoying information (spreading of viruses, rumors, online negative behaviors, and cyberbullying). Existing methods such as local centrality measures like degree centrality are less effective, and global measures like closeness and betweenness centrality could better identify influential spreaders but they have some limitations. In this paper, we propose the HybridRank algorithm using a new hybrid centrality measure for detecting a set of influential spreaders using the topological features of the network. We use the SIR spreading model for simulating the spreading processes in networks to evaluate the performance of our algorithm. Empirical experiments are conducted on real and artificial networks, and the results show that the spreaders identified by our approach are more influential than several benchmarks.

## Introduction

Spreading processes are ubiquitous in different complex systems. It occurs in a plethora of applications and domains, ranging from the spread of news and ideas to the diffusion of influence and social movements and from the outbreak of a disease to the promotion of commercial products. The interactions among the different entities of the network are responsible for the formation of the pathways, and for the definition of the topological properties of the entities; that widely affect the spreading phenomena in the networks. Understanding and controlling the spreading processes in complex networks has paid a great attention in the last decades: For example, promoting a new idea or new product in a network in order to be adopted by a large fraction of individuals. The word of mouth effect is the key behind the viral marketing, i.e. that individuals that have already adopted the idea will recommend it to their friends and so on. The problem of choosing key nodes as source spreaders to achieve the maximum scale of spreading is defined as influence maximization problem^1^. The basic question to control the spreading process and maximize the influence is identifying the set of influential spreaders leading to a successful promotion campaign.

Up to now, many centrality measures were proposed for ranking nodes and identifying their spreading ability in complex networks. There are three types of well-known metrics: local metrics, global metrics, and random-walk metrics. Local metrics like degree centrality are simple but are less effective because they neglect the global structure of the network^2,3^. Global metrics as betweenness centrality and closeness centrality are well performing in the identification of the key nodes, but they are of high computational complexity^2,3^. They are often considered prohibitive for large-scale networks and it’s hard to get the complete network structure for the large-scale networks. The random walk metrics like PageRank^4^ and LeaderRank^5^ show significant performance in directed and undirected networks. The paper of Lü *et al*.^6^ reviewed the state of the art of different proposed methods and approaches dealing with detection of vital nodes in complex networks. Different methods were compared based on the nature of the network (directed, weighted, bipartite, etc…). Each reviewed method (LocalRank, LeaderRank, ClusterRank, PageRank, degree centrality…..) performances depend on the objective functions under consideration. The betweenness centrality performs well in hindering epidemic spreading while in the SIR process, the degree centrality can better identify influential spreaders when the spreading rate is very small and the eigenvector centrality performs better when the spreading rate is close to the epidemic threshold. Kitsak *et al*.^7^ put forward a fast node ranking method called k-shell decomposition for large-scale networks. They argued that the node influence should be determined by the location of the node in the network. The nodes in the core of the network identified by the largest k-shell value are more influential than those in the periphery of the network. In the paper of Liu *et al*. named ranking the spreading influence in complex networks, a new method is proposed for measuring the spreading influence of nodes of the same k-core value. The spreading influence is measured by computing the shortest path from the target node to the network core^8^. Liu *et al*. provide a new method for improving the k-shell centrality by removing the redundant links that leads to densely connect the core nodes but they have a low diffusion importance. The redundant links are identified by measuring the diffusion importance for each edge based on the number of out-leaving links of its both ends^9^. Wang *et al*. present a new method for evaluating the influence capability of nodes using k-shell iteration factor, it uses the iteration information of k-shell decomposition to distinguish the influence capability of nodes with the same k-shell value^10^. Al-garadi *et al*. propose a new improvement of k-core centrality based on interactions between users for online social networks. The link-weighting method suggests that the interactions between users are a significant factor in quantifying the spreading capability of users^11^. Chen *et al*. provide an effective and efficient ranking method called ClusterRank and show that the spreading process initiated from the highly clustered nodes would be more likely to confine in a local region^12^. Chen *et al*. propose a new centrality measure named local centrality less time consuming compared to others centralities. The proposed centrality considers the nearest and the next nearest neighbors. For each node, the local centrality is the sum of the number of the nearest and the next nearest neighbors of each of its adjacent neighbors^13^. Qian and Jun propose the hybrid degree centrality, that combine Modified Local Centrality (MLC) which measure node’s distal influence and degree centrality and considers the different ratios between the importance of near-source influence (DC) and distal influence (MLC) under different spreading probabilities, while the spreading probability affect the result of those centralities^14^. Liu *et al*. deal with a new centrality named neighbor distance centrality (nbd) based on degree centrality and considers that the importance of the node depends not only on their direct neighbors but also on its neighbors of order 2 and 3^15^. The authors in^16^ provide a novel method to identify multiple spreaders from communities in a balanced way using the Red-black tree. The network is first divided into a great many super nodes using the blondel method and then k spreaders are selected from these super nodes. It takes a non-visited super node with maximal size from the red-black tree. Then, the most influential node is selected from the super node as a spreader according to a degree centrality index. A new family of H-indices for measuring the node importance is proposed in^17^. The H-indices are degree, H-index and coreness centrality that will be related in this work where degree, H-index (defined to be the maximum value h such that there exists at least h neighbors of degree no less than h.) and coreness are the initial, intermediate and steady states of the sequences, respectively. Zhang *et al*. propose VoteRank algorithm that measures for each node its ability of voting, the node getting most votes from its neighbors is selected as influential and it doesn’t participate in subsequent voting and the voting ability of its neighbors will decrease^18^. Wang *et al*. provide a new extension of the DegreeDiscount method named GeneralizedDegreeDiscount. In the proposed method, the status of a node is defined as its probability of not being influenced by any of its neighbors, and the index generalized discounted degree of one node is measured by the expected number of nodes that could influence^19^. The authors of^20^ provide a new method for influence maximization using optimal percolation in complex networks. In the beginning, all the nodes of the network are considered. Then, the node with the highest Collective Influence is removed from the network and the degree of their neighbors is decreased by 1. This process is repeated until the giant component of the network is zero. Wang *et al*. propose a new centrality namely efficiency centrality for the identification of influential nodes in networks based on network efficiency. In this method, the efficiency centrality of nodes is computed by analyzing the efficiency of the network before and after removing the node from the network^21^. Liu *et al*. propose the dynamic-sensitive centrality for locating influential nodes in networks by integrating topological features and dynamical properties. The spreading influence of a node at t is defined by the sum of infected probabilities when *i* is initially infected. The result of this method depends on the infection rate to be selected^22^.

Even if designing an effective method to evaluate the node spreading ability and detecting the influential spreaders in the networks has been addressed in several researches, however, it is still a large challenge up to now. In this paper, we propose a new method named HybridRank to detect the influential spreaders in the network using the topological features of the network. Our method can be split into two sections. First, we provide a new hybrid centrality for identifying the influential nodes of the network, and secondly we select a set of the influential spreaders, by interacting all together we maximize the spreading of influence. Our method is tested on four real networks, and the efficiency of our method is assessed using the SIR (Susceptible, Infected, Recovered).

The paper is organized as follows. Section 2 begins with a brief overview and definition of previous centrality measures. In section 3, we propose the HybridRank algorithm. In section 4, numerical examples in four real networks are illustrated to show the effectiveness and the performance of the proposed algorithm. Finally, conclusion and perspectives are presented in section 5.

## Centrality Measures

The centrality measures aims for identifying the “most important” nodes in a social network. They are used for understanding the power and the social influence in a network. The importance of node depends on diverse parameters such the direction in the graph, the connectivity and the nature of measurement of the entire network where the variety of the proposed measures^23–25^. Linton Freeman proposes the most important contributions for the analysis of social networks.

### Degree Centrality

It is defined as the number of links incident upon a vertex which means the number of edges a vertex has. For a graph G: = (V, E) with *n* vertices, the degree centrality C_d_(i, g) for vertex is:

1 $${C}_{d}(i,\,g)=\frac{{d}_{i}(g)}{n-1}=\frac{|{N}_{i}(g)|}{n-1}$$

### Betweenness Centrality

Vertices have higher betweenness if they occur on many shortest paths between other vertices. For a graph G: = (V, E) with *n* vertices, the betweenness C_b_(i, g) for vertex is computed as follows:

For each pair of vertices (v, w):

1. Compute all shortest paths between those vertices.
2. Define the fraction of shortest paths passing through the studied vertex i.e. vertex v.
3. Sum this fraction over all pairs of vertices (s, t).

The betweenness centrality is:

2 $${C}_{b}(i,\,g)=\frac{2}{(n-1)(n-2)}\sum _{k\ne j,i\notin \{j,k]}\frac{{P}_{i}(kj)}{P(kj)}$$

with: $\frac{{P}_{i}(kj)}{P(kj)}\,$is the probability that *i* falls on a randomly selected geodesic connecting *k* and *j*.

### Closeness Centrality

A vertex has higher closeness centrality if it is shallow to other vertices of the network, i.e. if it has short geodesic distances to other vertices. Closeness centrality is usually positively associated with other measures such as degree because it gives higher values to more central vertices, i.e. those with shortest-path length^26^.

The closeness centrality is:

3 $${C}_{c}(i,g)=\frac{n-1}{{\sum }_{i\ne j}d(i,\,j;\,g)}$$

where: *d(i, j; g)* is the geodesic distance between *i* and *j*.

## Methods and Materials

### Proposed Algorithm

Locating influential nodes in networks is a challenging task of huge importance because of its applications in complicated networks, marketing and advertisement, ranking web pages and scientists publications etc. Several methods are elaborated for the detection of these influential spreaders in networks. In a complex network, when a spreading origins from a single node, the final affected population depends much on the importance of the spreading origin. In this paper, the HybridRank algorithm will be presented to deal with the problem of influence maximization. In HybridRank algorithm, the main idea is to define a set of influential spreaders based on hybrid centrality; by interacting all together we maximize the spread of influence. Thus, our approach can be split to two points: (1) the identification of influential spreaders using hybrid centrality, and (2) the identification of a set of spreaders nodes that are susceptible to maximize the dissemination of influence by acting all together. The details of our algorithm are described as follows.

#### Step 1: Detection of influential nodes using hybrid centrality

Assume a social network that is modeled as a graph G = (V, E), with V being the vertex set. Each vertex in G represents an element in the dataset. *|V|* represents the number of vertices in G (or elements in the dataset). *E* is the edge set. Each edge represents a relationship between a pair of elements. *n* = |V| represents the number of network nodes and *m* = |E|, the number of edges. The network structure is represented as an adjacency matrix *A* = {*a*_*ij*_} and *a*_*ij*_ ∈ R, where *a*_*ij*_ = 1, if a link exists between nodes *i* and *j*, otherwise *a*_*ij*_ = 0.

The proposed hybrid centrality takes advantage from the global topology of a general network with no specific structure. As claimed by Kitsak *et al*.^7^, the location of a node determines its influence capability. Therefore, the nodes located in the core of network tend to be highly important than those in the periphery. Hence, the k-shell decomposition centrality method that decomposes the network into hierarchically structured shells from the core to the periphery. The k-shell decomposition method starts by removing all nodes with degree *k* = 1 and their links from the network. After removing all nodes with *k* = 1, there may appear some nodes with only one link left. We also remove these nodes until there is no node with one link left in the network. The removed nodes are assigned with an index *k*_*s*_ = 1. Next, nodes with degree *k* ≤ 2 are removed in a similar way and assigned an index *k*_*s*_ = 2. This pruning process continues removing higher shells until all nodes are removed. As a result, each node is assigned a *k*_*s*_ index, which is considered as the coreness of the node.

In this paper, a new improvement of the coreness of node is presented. The improved coreness of a node is equals to the coreness of its neighbors (Eq. 4). Thus, each node’s coreness depends of the *k* index of its adjacent nodes, i.e. the node is highly located (central) if their immediate neighbors are highly located (central).

4 $${\rm{ICC}}({\rm{v}})=\sum _{{\rm{u}}\in {\rm{\Gamma }}({\rm{v}})}{\rm{C}}({\rm{u}})$$

With (v) is the neighborhood of the node v.

Another centrality measures that is increasingly popular is the eigenvector centrality. It is a positive multiple of the sum of adjacent centralities^27^, and is based on the philosophy that a node is more central if its neighbors are also highly central. Because eigenvector centrality is proportional to an individual’s neighbors’ centralities^28–30^, more influential individuals will be more connected with other influential individuals.

5 $$Ax=\lambda x$$

With: *A* is the adjacency matrix of the network and *λ* is the eigenvalue.

In this paper, we present a new measure of centrality named hybrid centrality. The hybrid centrality is based on the previous cited centralities, i.e. the improved coreness centrality (ICC) and the eigenvector centrality (EC). Our proposed method is used to analyze the global features of nodes, and results are used to compute their global diversity. The hybrid centrality of a node v is defined as follows:

6 $$HC(v)=ICC(v)\ast EC(v)$$

With *ICC(v)* is the Improved Coreness Centrality of node v, and *EC(v)* is the eigenvector Centrality of node v.

The nodes are ranked based on their hybrid centrality (Eq. 6). The first influential spreader is the node with the highest hybrid centrality.

#### Step 2: Identification of a set of spreaders nodes

As pointed out by Kitsak *et al*.^7^, the propagation range would be improved greatly if any two selected spreaders are disconnected comparing with simply selecting nodes with maximum degree or k-shell value one by one. The previous step combines the improved version of k-core centrality and the eigenvector centrality. The idea behind combining those two centralities is that both of them consider a node as central if it is connected to other central nodes; i.e. the selected spreaders will infect their neighbors that are also powerful and influential; and in their turns; they will infect their neighborhood. In order to maximize the spread of influence, we avoid the selection of the adjacent neighbors when selecting the set of source spreaders from the ranked list. Based on those assumptions, the separation of spreaders nodes could accelerate the information dissemination and the selection of remote nodes can affect as many nodes as possible. For that, we will neglect the adjacent neighbors when selecting the set of source spreaders from the ranked list to maximize the spread of influence. So, after a node is elected as influential spreader, the selection probability of its neighbors will decrease. For that purpose, after the first spreader is selected, their adjacent neighbors will be eliminated from the ranked list. And the second spreader will be the node with the highest hybrid centrality in the remained ranked list.

HybridRank algorithm can be used to choose top-*k* influential spreaders in both undirected and directed networks. In directed network, if there is a link from node *u* to node *v*, *u* is the in-neighbor of *v*, and correspondingly, *v* is the out-neighbor of *u*. In this paper, a link from node *u* to *v* indicates that *v* receives information from *u*. In HybridRank version for directed networks, the identification of a set of source spreaders is based on in/out neighbors. Only the adjacent neighbors that receive influence from the spreaders will be deleted from the ranked list.

### SIR model

In this paper, we use the SIR epidemic model with limited contact to evaluate our method. In SIR model, each node of the network is in one of the three states: Susceptible (*S*) represents the individuals susceptible to be infected but not yet infected; Infected *(I*) denotes individuals that have been infected and are able to pass the disease to their susceptible neighbors with probability *β*; and Recovered (*R*) depicts individuals who are infected but have recovered with probability *γ*, and those nodes will never be infected again. The process terminates if there isn’t any infected node in network. In this paper, we set *γ* = 1 for generality. The real spreading ability initiated from node *i* is denoted as σ(i) by counting the number of recovered nodes over 100 simulations. We set the value of infection probability *β* to be slightly larger than the epidemic threshold ${\beta }_{th}\approx \frac{ < {\rm{k}} > }{ < {k}^{2} > }$of the network, where <*k*> and <*k*^2^> represent the average degree and the second order average degree, respectively^31^.

### Performance metrics

#### Kendall tau

Kendall tau coefficient^32,33^ is used to rank the real spreading ability of nodes referring to its spreading influence. It measures the correlation between the ranking method list and the one generated by the SIR model. The Kendall’s tau coefficient considers a set of joint observations from two random variables X and Y. Any pair of observation (*x*_*i*_, *y*_*i*_) and (*x*_*j*_, *y*_*j*_) are said to be concordant if the ranks for both elements agree: that is, if both *x*_*i*_ > *x*_*j*_ and *y*_*i*_ > *y*_*j*_ or if both *x*_*i*_ < *x*_*j*_ and *y*_*i*_ < *y*_*j*_. They are said to be discordant if *x*_*i*_ > *x*_*j*_ and *y*_*i*_ < *y*_*j*_ or if *x*_*i*_ < *x*_*j*_ and *y*_*i*_ > *y*_*j*_. It is defined as follows:

7 $${\rm{\tau }}({{\rm{L}}}_{1},\,{{\rm{L}}}_{2})=\frac{{{\rm{n}}}_{{\rm{c}}}-{{\rm{n}}}_{{\rm{d}}}}{\frac{1}{2}{\rm{n}}\,({\rm{n}}-1)}$$

where *L*_1_ and *L*_2_ are two different ranking with *n* elements, *n*_*c*_ and *n*_*d*_ represent the number of concordant and discordant pairs, respectively.

#### Infected scale

In order to compare the spread using different methods, we use the infected scale at time *t* which is introduced as follows:

8 $$F(t)=\frac{{n}_{I(t)}+{n}_{R(t)}}{n}$$

where *n* is the number of nodes of network, ${n}_{I(t)}and\,{n}_{R(t)}\,$are the number of infected and recovered nodes at time t respectively.

#### Final Infected scale

$F({t}_{c})$ is used to investigate the final scale of affected nodes.

9 $$F({t}_{c})=\frac{\,{n}_{R({t}_{c})}}{n}$$

where ${n}_{R({t}_{c})}\,$is the number of recovered nodes when spread process achieving steady state.

#### Shortest path length

The average shortest path length ${L}_{s}$ is used between each pair of source spreaders *S* to evaluate the structural properties among the selected spreaders.

10 $${L}_{s}=\frac{1}{|S|(|S|-1)}\sum _{\begin{array}{c}u,v\in S\\ u\ne v\end{array}}{l}_{u,v}$$

where ${l}_{u,v}$ is the length of the shortest path from node *u* to *v*.

### Data description

To ensure the efficiency and the performance of our proposed method, we assessed our method using both real networks and artificial networks. The artificial networks include networks generated by the Watts-Strogatz small-world network model (ws)^34^ of 1000 nodes and 5000 edges and the Barabàsi-Albert network model (BA)^35^ formed by 1000 nodes and 2994 edges. These networks are undirected and unweighted. As shown in Table 1, the four real networks include:

- Cond-mat is an undirected network of 23133 nodes and 93497 edges. It represents Arxiv COND-MAT (Condense Matter Physics) collaboration network is from the e-print arXiv and covers scientific collaborations between authors papers submitted to Condense Matter category. If an author *i* co-authored a paper with author *j*, the graph contains an undirected edge from *i* to *j*. If the paper is co-authored by k authors this generates a completely connected subgraph on k nodes^36^.
- Dblp network provides a comprehensive list of research papers in computer science of the DBLP bibliography. A co-authorship network is constructed based on the papers that gather authors. Two authors are connected if they publish at least one paper together. This network contains 317080 nodes and 1 million edges^37^.
- The Epinions directed network depicts who-trust-whom in the online social network of a general consumer review site Epinions.com. It is formed of 75879 nodes and 508837 edges^38^.
- Wiki-vote is also a directed network of 7115 nodes and 103689 edges that contains all the Wikipedia voting data from the inception of Wikipedia till January 2008. Nodes in the network represent Wikipedia users and a directed edge from node *i* to node *j* represent that user i voted on user j^39^.

**Table 1:** Topological features of the four real networks.

Datasets	*n*	*M*	*k*_*max*_	<*k*>	<*k*^*2*^>	<*cc*>	*β*_*th*_
Cond-Mat	23133	93497	281	8.083431	178.6619	0.6334	0.045
Dblp	317080	1049866	343	6.62089	144.0063	0.6324	0.045
Epinions	75879	508837	1801	6.7059	721.8229	0.1378	0.009
Wiki-vote	7115	103689	893	14.5733	1999.905	0.1409	0.007

*n* and *m* are the total number of nodes and edges, respectively. **<***k*> is the average degree for undirected networks or the average out-degree for directed networks. *k*_*max*_ is the maximum degree for undirected networks or the maximum out-degree for directed networks. <*cc*> is the average clustering coefficient and *β*_*th*_ is the epidemic threshold, defined as *β*_*th*_ $\approx \frac{ < {\boldsymbol{k}} > }{ < {{\boldsymbol{k}}}^{2} > }$.

## Results and Discussion

**Figure 1:**
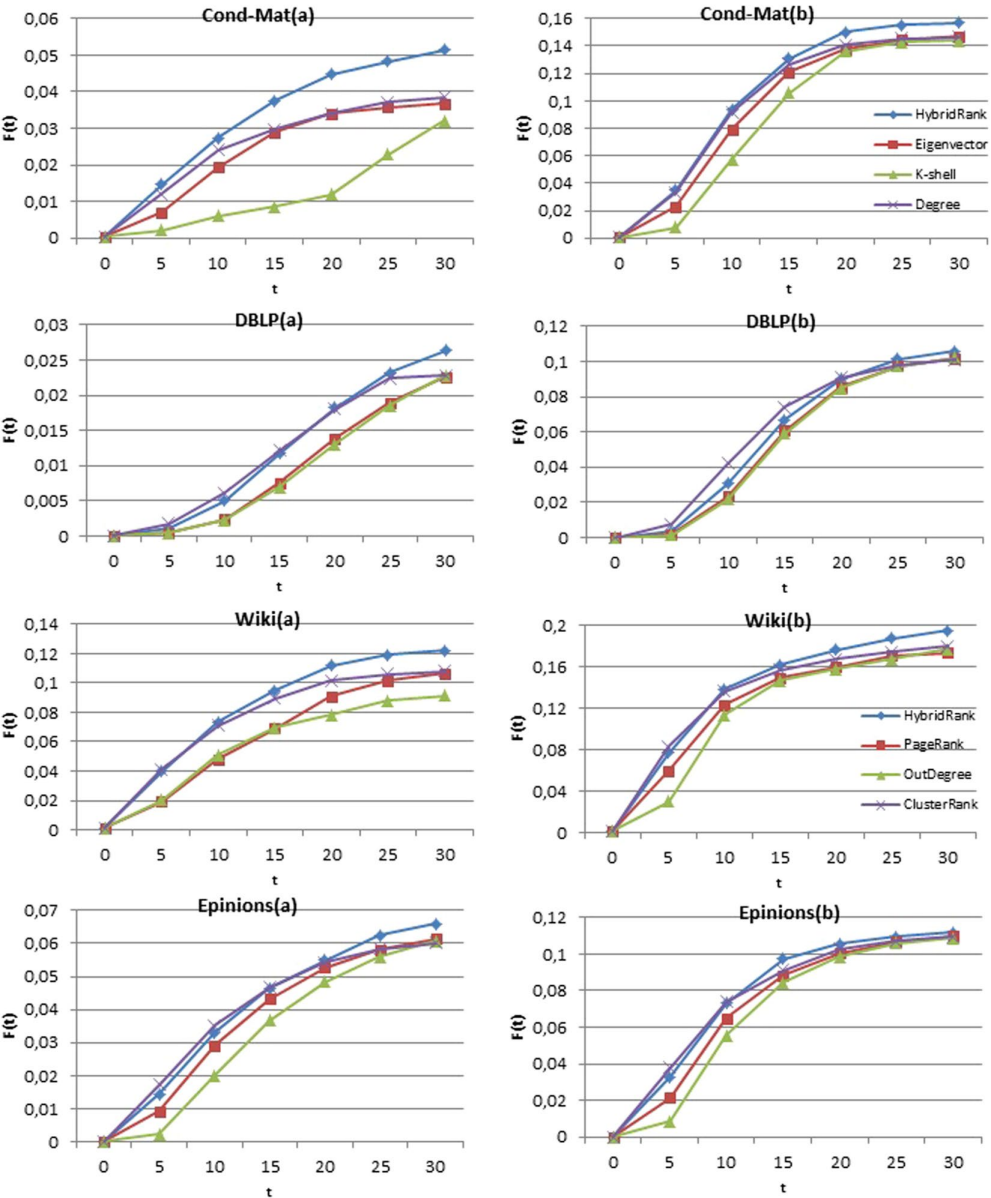
The affected scale F(t) for the four networks under different scale of infected rate β for different methods. In (**a**) β = 0.06 and in (**b**) β = 0.1 and γ = 1.

**Figure 2:**
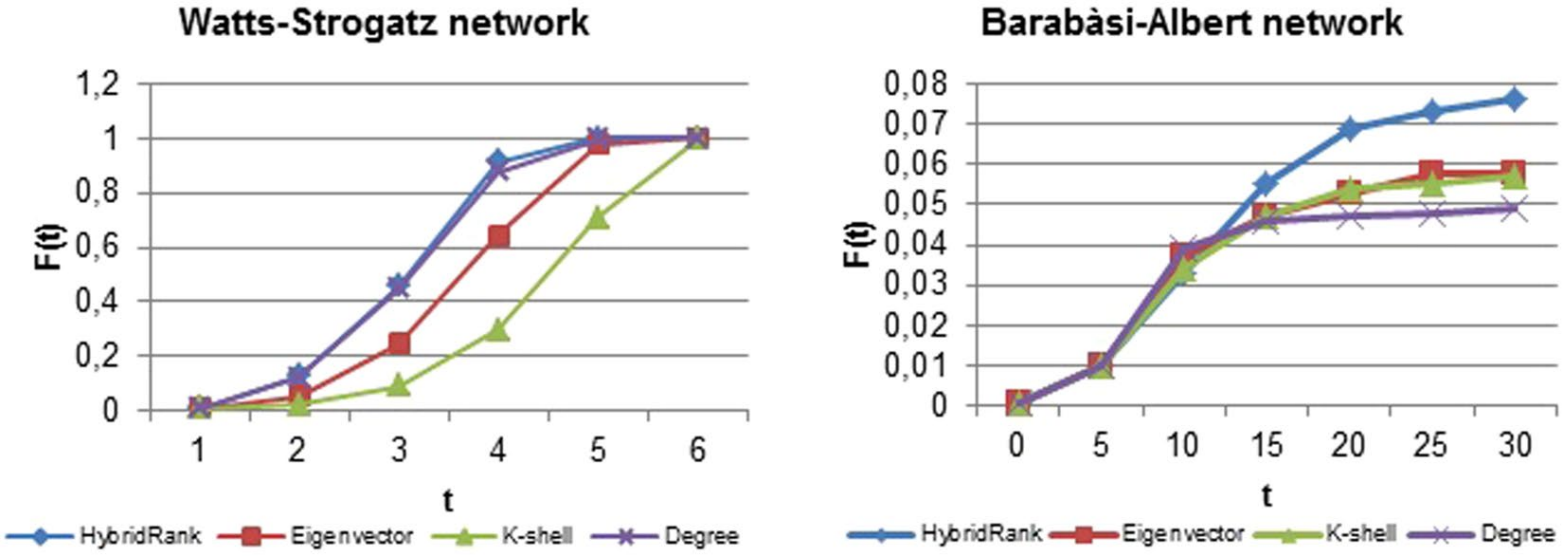
The affected scale F(t) for artificial networks with the infected rate *β* $\approx \frac{ < {\boldsymbol{k}} > }{ < {{\boldsymbol{k}}}^{2} > }$ and γ = 1 for different methods.

The performance of HybridRank algorithm compared to other methods is evaluated using different metrics mentioned before on both artificial and real networks. In each implementation, a fraction of the nodes is selected as source spreaders, and the information spreads according to the SIR process described above. For each method, the SIR process is repeated many times to ensure the stability of the results. Figure 1 shows the infected scale *F(t)* on four real networks (directed and undirected) where $p=\frac{10}{n}$ is the ratio of the number of source spreaders and *n* is the number of the nodes in the network. The results shown in (Fig. 1) are obtained using different range of infected rate *β* = 0.06 and *β* = 0.1 and *γ* = 1 for different methods. Besides the real networks, we also compare the result of our algorithm using artificial networks. Figure 2 represents the affected scale F(t) using different methods for the barabàsi-albert network and the watts-strogatz network where β = 0.09 and β = 0.1 successively and γ = 1. In the case of undirected networks, the result of our method HybridRank is compared to Eigenvector, K-shell decomposition and degree methods. For the directed methods, our algorithm HybridRank is compared to PageRank, OutDegree and ClusterRank^12^ algorithms. In our case, we set the *t* = 30 for further investigation, because the spreading in the early stages is more important. Using the source spreaders obtained by the HybridRank algorithm, it can be observed from (Figs 1 and 2) that source spreaders provided using HybridRank algorithm can affect larger scale compared to other methods even if the spread is smaller in primary steps. It is due to the set of selected source spreaders. The selected set of spreaders of Degree method have more connections; which explains the increase of the infected scale *F(t*) in primary steps and its decrease by the end. While in the HybridRank algorithm, the set of selected spreaders is not based only in the highest hybrid centrality, but also in the connections between spreaders, i.e. the selected spreaders shouldn’t get directed links even if the hybrid centrality of the chosen spreader decreases.

**Figure 3:**
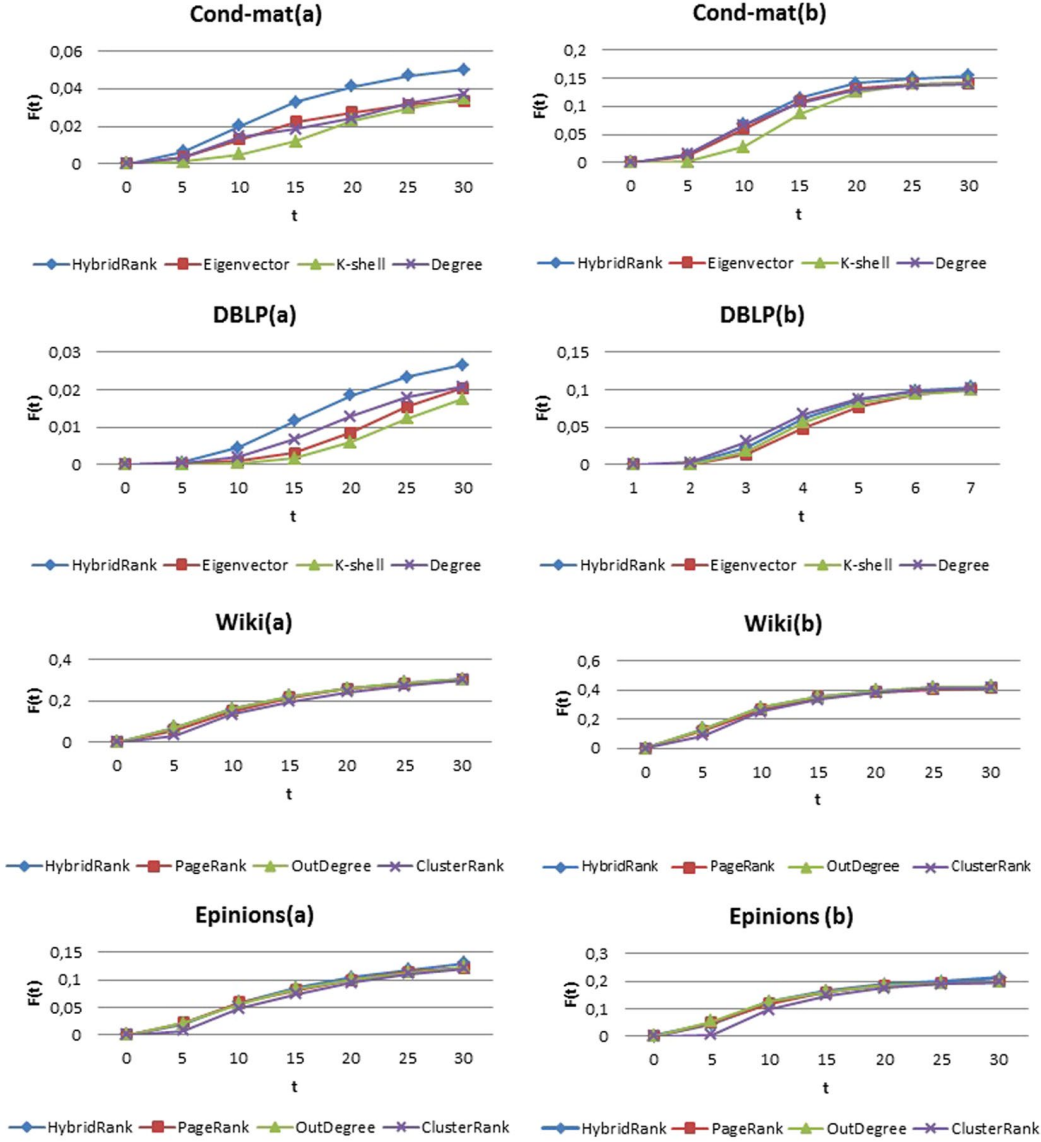
The affected scale F(t) for the four networks under different scale of infected rate β using one selected source spreader. In (**a**) β = 0.06 and in (**b**) β = 0.1 and γ = 1.

We have measured the spread of influence without neglecting the neighborhood using one selected spreader. Figure 3 shows the affected rate of nodes in range of t [0, 30] using one single node as source spreader for the four datasets. As shown in the figure, the HybridRank algorithm provides a high number of infected nodes compared to others methods. While, for the Wiki network, the HybridRank algorithm and OutDegree method provide the same result, because both methods select the same node as single spreader, i.e. the node with the high centrality in the ranked list.

Table 2 shows the final affected scale *F(t*_*c*_) of top-10 source spreaders with *β* is the infection rate, defined as $\beta =\frac{1}{ < k > }$ for undirected networks and $\beta =\frac{1}{ < {k}^{out} > }$ for directed networks, and the recovery rate *γ* = *0.8*. It’s obvious that HybridRank can achieve wider final affected scale *F(t*_*c*_) than other methods under same number of source spreaders. The final affected scale *F(t*_*c*_*)* is not only determined by the influence of source spreaders, but also by their location. For this reason, hybrid centrality can dig out single influential spreader effectively, but perform poorly on selecting set of source spreaders by only choosing those with the highest hybrid centrality. To overcome this limitation, as it was cited in our method description, our HybridRank algorithm chooses the group of source spreaders such as two chosen spreaders are not directly linked. Once the first spreader with highest Hybrid Centrality is selected, their neighbor will be not selected and eliminated from the list of ranked nodes.

**Table 2:** The final scale of affected nodes *F(t*_*c*_*)* for the four real networks in different algorithms averaging over 100 simulations.

Datasets	Algorithms	Final affected rate	Time steps	N	m	β	P
Cond-Mat	HybridRank	**0.2743008**	36.4	23133	93497	0.124	0.0004
	K-shell Rank	0.2691739	37				
	EigenvectorRank	0.263096	36.6				
	DegreeRank	0.2679635	32.4				
Dblp	HybridRank	**0.2679961**	42.6	317080	1049866	0.151	0.00003
	K-shell Rank	0.2679305	46.2				
	EigenvectorRank	0.2673779	44				
	DegreeRank	0.2665107	39.8				
Epinions	HybridRank	**0.1812517**	29.8	75879	508837	0.149	0.0001
	ClusterRank	0.178529	33.8				
	PageRank	0.1789006	37.2				
	OutDegreeRank	0.1787477	33				
Wiki-vote	HybridRank	**0.1602811**	39.8	7115	103689	0.068	0.0015
	ClusterRank	0.122052	26.4				
	PageRank	0.1519325	31.6				
	OutDegreeRank	0.1496275	28.4				
BA	HybridRank	**0.3822**	18.4	1000	2994	0.167	0.01
	K-shell Rank	0.348	16.2				
	EigenvectorRank	0.3208	19				
	DegreeRank	0.3222	17.8				
WS	HybridRank	**0.0938**	16.8	1000	5000	0.1	0.01
	K-shell Rank	0.0218	8				
	EigenvectorRank	0.0354	9.8				
	DegreeRank	0.0484	11.2				

*n* and *m* are the total number of nodes and edges, respectively. *p* is the ratio of the number of source spreaders and *β* is the infection rate, defined as *β* $=\,\frac{1}{ < {\boldsymbol{k}} > }$ and *γ* = *0.8* is the recovery rate.

**Figure 4:**
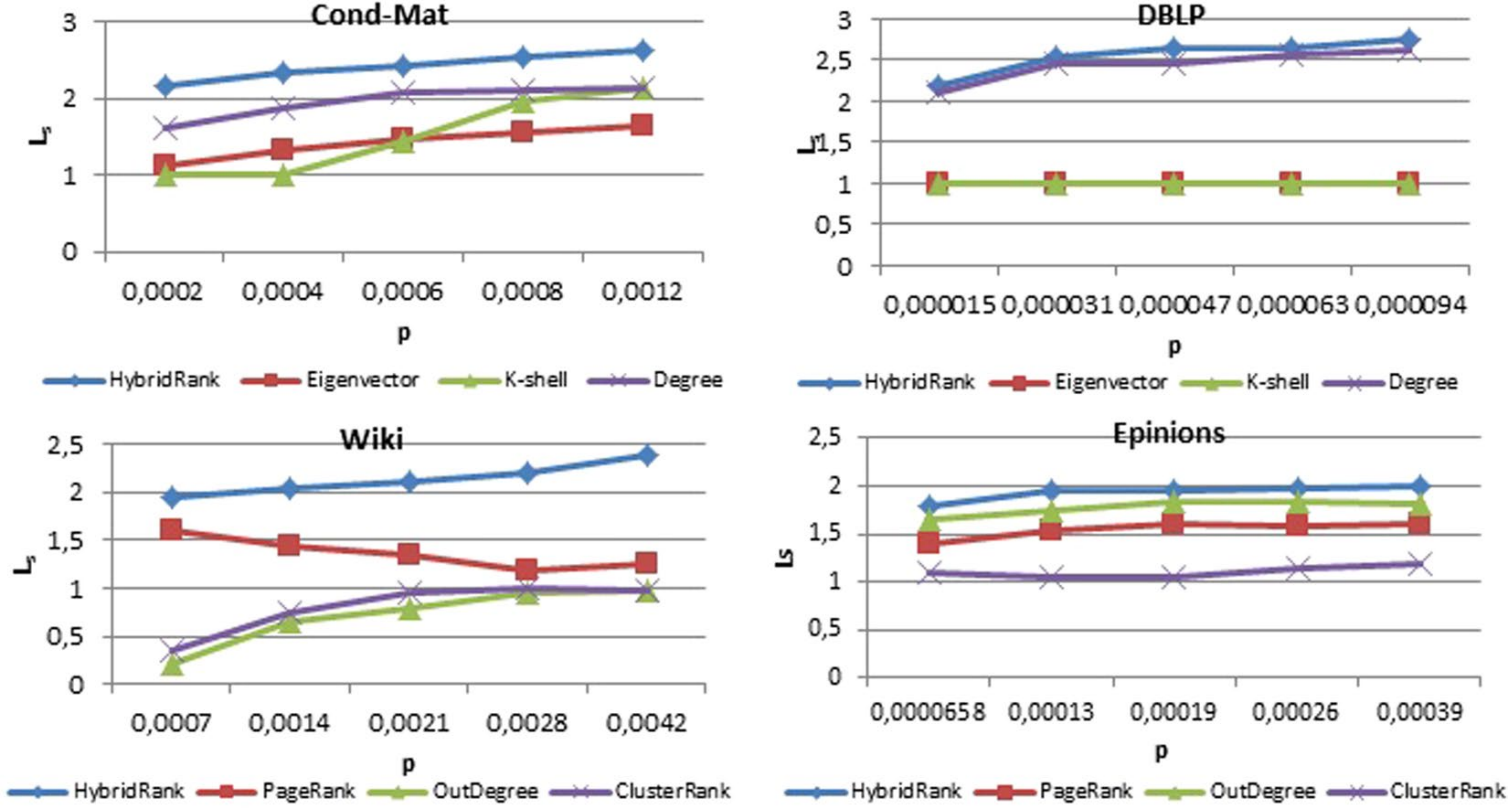
The average shortest path length *L*_*s*_ under different scale of source spreaders for different benchmark methods.

To ensure that the selected source influential spreaders obtained using HybridRank algorithm are more scattered than other methods, the average shortest path length *L*_*s*_ of HybridRank and other algorithms are compared. Figure 4 shows the average of shortest path length *L*_*s*_ of source spreaders selected by different methods under different scale of source spreaders $p=(\frac{5}{n},\,\frac{10}{n},\,\frac{15}{n},\,\frac{20}{n},\,\frac{30}{n})\,$. As demonstrated in Fig. 4, the selected influential spreaders obtained using HybridRank algorithm have larger *L*_*s*_ than those obtained by other methods for both real and artificial networks.

For the Cond-mat, Dblp, Wiki-Vote and Epinions networks, the Kendall’s tau correlation values for different methods are shown in Table 3. The real spreading ability of nodes is measured using the SIR model with *β*_*th*_ $\approx \frac{ < k > }{ < {k}^{2} > }$ is the epidemic threshold and the recovery rate *γ* = 1 averaging over 100 simulations. For the undirected networks, the ranked list (*σ*) of top-10 of network nodes obtained using the SIR process is compared with the HybridRank, eigenvector, degree and k-shell decomposition ranked lists. While for the directed networks, the ranked list *σ* is compared to our HybridRank, ClusterRank, PageRank and Outdegree ranked lists. The indices of correlation in directed networks obtained using the HybridRank algorithm are higher than others methods. While in Cond-mat network, the indice of HybridRank is smaller than degreeRank and k-shell due to the degree of nodes and the two ranked lists obtained using degree and k-shell centralities are specially based on degree, therefore their spreading ability is higher; i.e. both degree centrality and real spreading ability lists are ranking their nodes based on their degree. In fact, in the real spreading ability list, a node could infect a higher number of nodes if it is highly connected i.e. the ranking is determined by the degree, the same as the degree centrality. Therefore, the correlation between the real spreading ability list and the degree centrality list is higher in the undirected networks because both methods ranked the nodes based on degree.

**Table 3:** The Kendall’s tau correlation of different measures compared to the ranked list of SIR.

Datasets	τ(k_s_, σ)	τ(EC, σ)	τ(HR, σ)	τ(DC, σ)	τ(CR, σ)	τ(PR, σ)
Cond-Mat	0.28	0.22	0.26	0.31	—	—
DBLP	−0.02	−0.06	0.11	0.28	—	—
Wiki-Vote	—	—	0.56	0.33	0.04	0.17
Epinions	—	—	0.11	−0.02	−0.24	−0.33

### Computational efficiency

The HybridRank algorithm has two steps: the hybrid centrality calculation and the identification of a set of influential spreaders in the networks. The hybrid centrality is composed from the eigenvector centrality of complexity O(|V| + |E|) with |V| is the number of vertices and |*E*| is the number of edges, and the complexity of the improved coreness centrality (ICC) of O(|E|). The complexity of the second step is depends on the complexity of the neighborhood function. While the complexity of the neighborhood of graph nodes is O(|V|*d*o) with *d* is the average degree of the network and *o* is the order of neighborhood. If we want to select *m* influential spreaders, the algorithm should be run for *m* rounds. Therefore, the complexity is O(m*<k>*o) where <*k*> is the average node of nodes of the network and *o* is 1 because we are looking for neighbors of order 1. Thus, the complexity is O(m* <k>). Then, the total complexity of the HybridRank algorithm is: O(|V| + |E| + m <k>). In many networks, the average degree <*k*> is less than the number of nodes <*k*> ≪ |*V*|. Thus, the final complexity is O(|V| + |E|).

## Conclusion

In this paper, we propose a novel HybridRank algorithm to select k influential source spreaders based on our proposed hybrid centrality. In our method, the set of influential spreaders is not chosen only based on the hybrid centrality, but the set of selected source spreaders should not be adjacent to maximize the spread. The performance of our method is evaluated in four real networks (directed and undirected) under the Susceptible-Infected-Recovered (SIR). Results show that our proposed algorithm outperforms several benchmark methods using different metrics. As further work, the HybridRank algorithm will be used for community detection algorithm based on leaders/influentials nodes. The leaders/influential nodes will be the identifiers of the communities, and we will assign for each leader/influential node their similar nodes to form the communities.
